# Optimizing Social Support in Oncology with Digital Platforms

**DOI:** 10.2196/36258

**Published:** 2022-06-24

**Authors:** Dimos Katsaros, James Hawthorne, Jay Patel, Kaitlin Pothier, Timothy Aungst, Chris Franzese

**Affiliations:** 1 Matchstick LLC Boonton, NJ United States; 2 College of Pharmacy University of Rhode Island Kingston, RI United States; 3 Massachusetts College of Pharmacy and Health Sciences Worcester, MA United States

**Keywords:** social support, chronic disease, social networks, oncology, cancer, digital biomarkers, digital health, caregiver support

## Abstract

Increased cancer prevalence and survival rates coupled with earlier patient discharges from hospitals have created a greater need for social support. Cancer care is both short term and long term, requiring acute treatments, treatments for remission, and long-term screenings and treatment regimens. Health care systems are already overwhelmed and often struggle to provide social support systems for everyone. Caregivers are limited in number, and even when they are available, they often lack necessary information, skills, or resources to meet the needs of patients with cancer. The act of caregiving presents various challenges, and caregivers themselves often need social support as well. Despite these needs, most social support programs are targeted toward patients alone. Given the prevalence of cancer and known needs of these patients and their caregivers, the ability to identify those who need social support is crucial. Further, the scalability and overall availability of social support programs is vital for successful patient care. This paper establishes the benefits of social support for both patients and caregivers coping with cancer treatments, explores innovative ways of identifying patients who may need social support using digital tools, and reviews potential advantages of digital social support programs.

## Introduction

Life expectancy in the United States has risen over 66% in the past century due, in part, to medical advancements [1]. Importantly, the fastest growing segment includes those aged 65 years and over, with the number of older adults worldwide in 2015 projected to more than double by 2050 [2]. Extended life expectancy has coincided with a steady rise in chronic disease, with incidence rates increasing by 7 to 8 million every 5 years for the past two decades [3].

Advancements in diagnostics and therapeutics have resulted in an increased number of cancer survivors [4]. Cancer is no longer strictly an acute disease; over the next decade, nearly 40% of patients diagnosed with cancer are expected to live greater than or equal to 5 years after diagnosis [4]. With patients living longer, cancer treatment is now often chronic in nature, requiring ongoing care and support for long-term management [5]. Despite advancements in treatments, however, cancer remains a leading cause of both death and disability worldwide [6,7]. Notably, the reported figures from 2020 may be a gross underestimation due to the debilitating effects of the COVID-19 pandemic on cancer screenings, diagnoses, and treatments. As a result, the coming years may show an even greater uptick in cancer incidence and mortality rates on a global level [6].

Social restrictions during the COVID-19 pandemic have drawn attention to the impact of social support, and lack thereof, on mental health in the general population [8]. However, the particular importance of social support for patients with chronic disease has been long established. In this context, social support is multifaceted and consists of providing patients with emotional, information, or practical support. Social support may come from various sources, such as family, friends, partners, and health care professionals [9]. Social support that comes from casual interactions with family, friends, or peers is often called informal social support, while support that comes from professional services, such as a nurses, physicians, or social workers, is considered formal social support [10].

While formalized social support programs exist, their use is not widespread among patients with cancer. Commonly cited reasons for underuse of formal social support programs include lack of awareness of existence among patients, absence of recommendation from physicians, practical constraints, and financial barriers. Further, formal social support programs are generally used more frequently by White, female, and higher-educated individuals, leading to further underuse by minority groups [11].

With formal social support having many barriers to use, patients often turn to their social network for informal social support [11]. Informal social support has been shown to impact clinical outcomes, including morbidity and mortality, as well as psychological outcomes, such as mood, mental health, and quality of life (QoL) [12-19]. For many patients with cancer, however, casual social interactions are not enough and, in turn, many patients rely on their caregiver for these interactions. Increased cancer prevalence and survival rates coupled with earlier hospital discharges to conserve financial resources have created an even larger and more important role for caregivers [6,20]. However, caregivers often lack necessary information, skills, or resources to meet the needs of patients with cancer and often need to cope with the significant emotional burden associated with caring for such patients [20,21]. Thus, caregivers must also be considered when discussing the importance of social support in cancer.

Considering these factors, a newfound structure for social support that encompasses both patients and caregivers will be essential to ensure quality and scalability of cancer care. With potential increases in global cancer incidence, prevalence, and mortality in the coming decades, achieving adequate social support may require leveraging digital tools that can proactively meet patient and caregiver needs. This paper seeks to summarize the importance of social support in chronic disease, outline its different components, suggest the use of digital biomarkers to identify patients who need social support, establish the advantages of digital social support programs, and review currently available digital modalities of social support for patients and caregivers in the oncology space.

## Components of Social Support

While prior literature has defined social support in different ways, it is often comprised of three main facets: emotional, practical, and informational support [22-25]. These aspects of social support are summarized in Table 1. Emotional support focuses on expressing empathy and compassion to patients and caregivers, and fostering the sentiment that they are not alone in their experiences. Practical support fulfills the physical needs that patients or caregivers may have, such as transportation to the hospital or picking up medications. Informational support involves sharing education or tips about managing the disease state with patients or caregivers. As mentioned previously, social support can come via formal support programs or informal support from friends, families, or peers. While formal medical information should come from health care providers (HCPs), a patient’s peers can provide valuable information, such as what to expect with certain treatments, particularly if the peer has the same chronic disease. Patients are not medical experts, but they often have valuable personal experiences from coping with disease that many providers may not.

**Table 1:** Major aspects of social support.

Major aspects of social support	Description
Emotional and physiological	Empathy, encouragement, and reassurance
Practical	Support with physical needs of daily living
Informational	Personal experiences, tips, and facts

## Importance of Social Support in Patient Care

The clinical complexity of a cancer diagnosis poses a significant burden on patients. However, burden is not solely related to clinical complexity but is also a function of the dynamic interplay between personal, social, and clinical aspects of a patient’s experience [26]. Thus, even if therapeutics have favorable side effect profiles and minimal disruption to patients’ lives, the social aspect of a cancer diagnosis could pose a significant burden. A recent large-scale analysis found that nearly 40% of patients with chronic disease reported that they are unable to sustain their current investments of energy, time, and money into health care lifelong [27]. As patients with chronic diseases often have complex care plans that can be discouraging and difficult to manage independently, social support may play a crucial role in helping them be successful and feel less overwhelmed in trying to cope with their disease. Moreover, chronic disease is also associated with a higher risk of mental health conditions, such as depression and anxiety, which can limit patients’ activity levels and social interactions [28].

Given the known challenges associated with managing chronic conditions, several studies have sought to understand the potential benefits of providing social support services in this setting [10-14]. One study of older adults with diabetes found that greater social support was associated with less stress, lower rates of depression, and reduced risk of myocardial infarction. Further, social support was also associated with fewer impairments in activities of daily living (eg, eating, drinking, bathing, and dressing) and instrumental activities of daily living (eg, using the telephone, transportation, laundry, and finances) [13]. Another large-scale meta-analysis found that greater perceived social support had a protective impact on mortality of up to 66% [17]. The same analysis found that a greater social network, such as regularly seeing friends and family members, can reduce the risk of mortality up to 70% [17].

While once not considered a chronic disease, improvements in diagnostics and therapeutics have transformed cancer into a condition requiring long-term care and support [5]. Compared to other diseases, cancer may be far more demanding and may, therefore, require a higher degree of social support [29]. Patients with cancer and their caregivers often cope with convoluted treatment plans, regimens associated with significant toxicities, symptom and side effect management, high disease severity, higher care costs, and more stressful decision-making [29]. Along with physical and medical impacts, a cancer diagnosis often brings along a heavy emotional burden; the prevalence of both depression and anxiety are significantly higher in patients with cancer [30]. Prognosis, pain levels, body image disruption, and tumor- or treatment-related side effects can all heavily impact a patient’s psychological state [30].

The importance of social support for patients with cancer is well recognized, as current clinical guidelines recommend the incorporation of social support into oncology patients’ care plans to help mitigate the distress associated with their disease [31].

Patients with cancer commonly express a desire for social support, as one study found patients often requested companionship, empathy, and home care support. More specifically, companionship was requested by nearly half of patients [32]. Beyond just a desire for social support, numerous studies have specifically demonstrated the benefit of social support services for patients with cancer. In terms of emotional benefit, providing social support has been shown to lessen feelings of anxiety and depression associated with the disease [33,34]. Social support has also been shown to have tangible outcomes, including increased medication adherence. In a prospective study of women with breast cancer, greater social support during oral endocrine therapy initiation was associated with higher rates of adherence and fewer depressive symptoms [35]. The costs associated with medication nonadherence in the United States is estimated to be US $100 billion to $290 billion, and nonadherence to cancer therapies is reported to be significantly higher than that of other disease states [36]. Thus, improving medication adherence among patients with cancer could have significant implications for reducing health care costs.

Beyond medication adherence, social support has been associated with improved physical and emotional health, well-being, and overall survival in patients with cancer [37]. One meta-analysis of 87 studies found a 12% to 25% reduction in relative mortality in patients with high levels of perceived social support and large social networks as well as in those who were married [38]. Social networks can also have a significant impact on overall QoL. One study of over 3000 breast cancer survivors found that larger social networks were associated with statistically significant higher QoL. More specifically, the study found that “the availability of someone with whom to have fun, relax, and get one’s mind off things for a while” had the strongest association with QoL improvement [39]. Despite the established benefit, formal social support is not widely adopted in clinical practice. One study found that only 8% of patients reported attending a cancer support group, and almost 60% of patients did not know where to find a group [11]. While HCPs commonly recommend patient support programs, they often do not give direct recommendations on where to find such programs. Further, certain demographics, such as patients living in distant or rural areas, reported less use of social groups [11]. While support groups may be beneficial for such patients, the current infrastructure of physical support groups renders them impractical for many patients, and new approaches to provide social support are warranted.

## Social Support for Caregivers

Given the rise in cancer prevalence and desire for more convenient treatments, a significant proportion of cancer care has shifted to the outpatient setting [2,7,40]. New oncologic agents have facilitated this transition, as they are more targeted and less invasive, making outpatient administration feasible [40]. While this offers more freedom to patients, it may place a larger strain on caregivers who are often relied on by many cancer patients for logistical support, including assistance with treatments, home care, and other tasks of daily living. These caregivers are frequently family members or friends who are not paid for the services they provide [41]. Along with the difficulty of performing complex care tasks with little to no training, a heavy emotional burden is placed on the shoulders of these informal caregivers, which in and of itself has been associated with increased mortality risk. One study found that older adult spousal caregivers who were experiencing caregiver strain had a 63% increase in 4-year mortality compared to a control group matched for age and sex who did not provide caregiving [42].

Moreover, approximately 9% of informal caregivers report having nobody to talk to about private matters. This feeling of isolation is exacerbated in millennial caregivers, with 27% indicating that they are not satisfied with the quality of their social relationships, and with 2 out of 10 reporting that they do not see any of their friends in a given month [21]. Fewer social connections and lower satisfaction with social support also significantly predicted depressive symptoms among caregivers, especially women [21]. Further, caregivers who are socially isolated are at greater risk of experiencing difficulties with complex care [21]. Although social support interventions are available for caregivers of patients with cancer, an estimated one-third of caregivers fail to ask for support when they need it [43].

## Psychosocial Burden of Cancer

The psychosocial burden of cancer can be overwhelming, and many patients may not have a support system to help them cope with the emotional aspects of the disease. Unfortunately, psychosocial complications are common in patients with cancer [44]. Concerningly, many patients suffering from psychosocial comorbidities are not adequately treated. One study found that 73% of patients with cancer were not receiving potentially effective treatment to manage their depression, and only 5% were seeing a mental health professional [45].

Many of these patients with psychosocial burdens may not have access to informal social support, let alone formal social support. As cancer disproportionately affects the geriatric population, older adults often have a reduced network accessible for social support due to life cycle events, such as retirement, death of loved ones, and biological effects of aging (eg, new sensory impairments and worsening chronic illnesses) [46]. Social isolation and lack of perceived socio-emotional support can be detrimental to patient health, as studies have shown that social isolation can lead to reduced QoL and increased morbidity and mortality among patients with cancer [39,47,48].

These psychosocial burdens of cancer often go unaddressed but have a significant impact on the economic cost of cancer. One systematic review estimated the monetized lifetime psychosocial cost burden of cancer care in Canada, as measured by health-related QoL costs, to be CAD $427,753 to $528,769 (US $320,815 to $396,577), which represents approximately two-thirds of the economic cost of cancer [49]. Unlike clinical burden, psychosocial burden is less tangible and, thus, more difficult to identify, quantify, and formally diagnose [50]. Thus, patients with high needs for social support may not be identified early enough or, in some cases, at all. Moreover, there may be great interpersonal variation in psychosocial burden, even within patient populations with the same diagnosis. Identifying patients with high psychosocial burdens and proactively providing social support could improve the lives of patients and significantly decrease the economic burden associated with cancer.

## Digital Biomarkers to Identify Patients in Need of Social Support

Digital biomarkers are objective measurements of physiological, pathologic, or anatomic characteristics continuously collected outside the clinical environment via home-based connected devices [51]. Passively collecting data from patients’ mobile or wearable devices potentially offers a convenient and unobtrusive method to prospectively identify psychosocial burden and deliver tailored social support to the right patients at the right time. To our knowledge, digital biomarkers are not currently used to identify social support needs specific to cancer. However, they have been successfully used to identify depression, anxiety, and stress, all of which are common psychosocial complications of cancer [52-54].

One study examined the use of algorithms incorporating digital trace data, such as device location and phone usage, along with voice data from mobile devices to identify behavioral indicators of clinically validated symptoms of depression and posttraumatic stress disorder [52]. The behavioral indicators that were measured included depressed mood most the day, diminished interest or pleasure in all or most activities, fatigue or loss of energy, and avoidance of activities, places, or people. Digital biomarkers have also been used to identify mood disorders, such as major depressive disorder and bipolar disorder. Data on patient movement, captured over 2 weeks, was able to successfully predict diagnosis status of a mood disorder 89% of the time [53]. Another study combined passively collected data about patient movement, using accelerometers, and social contact, using calls and texts, over 2 weeks with machine learning models to predict social anxiety symptom severity [53]. Social anxiety is particularly important in the field of cancer, as it has been referred to as a hidden psychiatric comorbidity in patients with cancer [54]. While less specific, the use of smartwatches, rings, body patches, body scales, and vests can provide physiological proxies of the autonomic nervous stress response, such as resting heart rate, electrodermal activity, cortisol levels, and inflammatory cytokines [55]. Real-time tracking of these objective measures of stress could help identify patients who need social support and help providers monitor patients’ stress throughout therapy. Overall, given the high prevalence of mood disorders in patients with cancer, the ability to diagnose psychosocial comorbidities at scale using digital tools could be a crucial means to deliver both social and pharmacologic interventions. This approach could potentially identify patients in need sooner, alleviate the workload required to diagnose psychosocial burdens in current practice, and reduce associated health care use.

## The Role of Social Media Platforms in Social Support

Although support in the form of in-person interventions represents a significant portion of the social support provided, in-person programs may not be suitable for many patients. While in-person interventions have known benefits, accessibility to in-person social support groups is not always possible. Transportation, awareness of programs, the ongoing COVID-19 pandemic, or program availability could still hinder a patient’s ability to attend physical social support groups. Further, the growing number of patients with cancer is greatly outpacing the capacity for care of the current health care system [56]. The health care system may not be able to physically provide social support for all patients that need it. New social support methods must be flexible enough to allow broad patient and caregiver access despite these limitations.

Unlike physical social support programs, digital support programs offer the potential for continuous access from the comfort of a patient’s home without the presence of a physical HCP. While digital support programs may not have been broadly accessible a decade ago, the widespread adoption of computers and smartphones across almost every demographic in the United States enables access for the vast majority of patients with cancer [57]. Digital support programs have the potential to compensate for diminishing face-to-face physical interactions, while always providing an avenue for social support. Social media platforms have historically been used as outlets toward informal social support. Forum-style Facebook groups offer patients the opportunity to connect and form relationships with others who may have gone through or are going through similar experiences. Many platforms also allow users to form or join specific groups. These groups allow patients to find and interact with each other informally and in real time. Importantly, these platforms enable asynchronous interactions with other patients, giving them flexibility depending on when they want social support or can invest time into social support. Further, specific groups can be made within these platforms to support minority groups to overcome language or cultural barriers.

While social media platforms offer theoretical benefits, their impact on patients is highly variable. A systematic review found that while 48% of studies indicated benefits from social media platforms, 45% found the effects to be neutral, and 7% of studies actually suggested harm from their use [58]. Still, both patients with cancer and their caregivers have expressed that they wish to use social media for the purpose of both social and emotional support. In one study, caregivers specifically expressed that they wanted to form connections with other caregivers in similar situations with which to share experiences and information [59]. With that said, social media platforms also pose risks to patients and caregivers, including possible privacy and confidentiality concerns, no regulation to ensure that accurate medical information is conveyed, a propagation of negative health behaviors, and information overload [60]. Further, many groups on social media platforms may be hidden, hard to find, or private, which may make it difficult for all patients to benefit. A platform targeted for digital social support in oncology must protect health care information, increase awareness of social support, and improve accessibility to digital social support.

## Recommendations

With smartphones and wearable connected devices now ubiquitous, using these devices for data collection is easier than ever to employ from a technological standpoint. Still, collected data must also be systematically translated into useful information for clinicians. Simply providing biomarkers to already-burdened providers will likely not amount to advancements in care delivery. Further research is needed to identify specific algorithms encompassing particular sets of biomarkers that can accurately identify psychosocial burdens in patients with cancer, while limiting required interventions from HCPs.

Once those who require social support are identified, flexible programs must be made available without relying on particular channels (eg, in-person support groups), as these may isolate certain patients or caregivers. Creating both accessible and scalable social support programs will be crucial to accommodate the rise in cancer diagnosis and to decrease the psychosocial impact of cancer on patients and their caregivers. Physical social support programs have established benefits, but these programs also have limitations, such as transportation, pandemic-related concerns, fixed group meeting times, and finite availability. Digital platforms can avoid these limitations and increase availability and accessibility of social support, even among minority groups.

Despite promise, funding for such digital biomarkers to identify social support needs, as well as the associated digital platforms to deliver social support, may be challenging. The economic benefit of providing social support through these avenues is not currently well established, and it is unclear whether health care payers would support new digitally based programs. Targeted research that specifically identifies concrete clinical and economic value in digital social support programs to foster further development is needed.

## Conclusions

Despite the established benefits of social support programs, their use among patients with cancer is not widespread. Patients often do not seek social support when they need it or may not even know they need social support. Patients and the health care system alike are in desperate need of a more efficient method of identifying patients who need social support. Digital biomarkers collected via mobile or wearable devices offer an innovative, yet relatively facile, way of identifying a subset of patients that need social support.

Although the need for social support in patients with cancer is high, the current infrastructure to provide social support is largely underdeveloped and unable to accommodate patient needs for support. Health care systems are already struggling with capacity limitations to provide care for all patients. Tailored digital platforms can provide accessible social support, without straining the already-burdened health care system.

## Abbreviations

HCP: health care provider
QoL: quality of life
